# Impacts of In Situ Wheat Straw Incorporation Methods on Cadmium Behavior in Soil–Rice Systems

**DOI:** 10.3390/foods15122057

**Published:** 2026-06-06

**Authors:** Leilei Li, Zhengbo Peng, Cheng Wang, Guanzhou Luo, Yuanqing Shi, Ruhongji Liu, Mingming Hu, Chuanhai Shu, Hao Fu, Feijie Li, Xinghai Huang, Qin Liao, Zhonglin Wang, Zhiyuan Yang, Yongjian Sun, Zongkui Chen, Jun Ma

**Affiliations:** 1 Rice Research Institute, Sichuan Agricultural University, Chengdu 611130, China; 2 Crop Ecophysiology and Cultivation Key Laboratory of Sichuan Province, Chengdu 611130, China; 3 College of Natural Resources and Environment, Northwest A&F University, Yangling 712100, China; 4 Rong County Bureau of Agriculture and Rural Affairs, Zigong 643100, China

**Keywords:** cadmium (Cd) contamination, straw biochar incorporation, rice–wheat rotation, low-Cd-accumulating cultivar

## Abstract

Cadmium (Cd) contamination in paddy soils poses a severe threat to food safety. Although straw incorporation is a key approach to sustainable agriculture, the mechanisms underlying its regulatory effects on safe rice production in Cd-contaminated fields remain unclear. This field study, conducted at two Cd-contaminated sites with two rice cultivars differing in Cd accumulation (low-Cd ZLY8612 and high-Cd YXY2115), evaluated five wheat straw management practices: straw removal (CK), straw mulching (SM), straw incorporation (SI), straw incorporation with organic fertilizer (SOI), and straw-derived biochar incorporation (SBI). The primary findings revealed that SOI and SBI could effectively reduce Cd availability and promoted Cd transformation to residual fractions, with SBI showing superior immobilization effects. SBI also enriched beneficial taxa (*Bacillus, Sphingomonas,* and *Flavisolibacter*), increased *Proteobacteria*, and reduced *Chloroflexi* and *Acidobacteriota*. All treatments enhanced rice yield; however, only SBI reduced grain Cd in the high-Cd cultivar to <0.2 mg/kg with high-Cd soil. Collectively, the combined application of straw-derived biochar incorporation and low-Cd-accumulating rice cultivar is a reliable and recommendable agronomic strategy for safe grain production and sustainable straw recycling in Cd-contaminated rice–wheat rotation fields.

## 1. Introduction

Food security and food safety are global challenges, and soil health serves as their fundamental safeguard. Rice is the dominant staple crop in China, and its safe production is indispensable to national food security [1]. However, soil cadmium (Cd) contamination has become increasingly severe, primarily due to atmospheric deposition, wastewater irrigation, excessive phosphorus fertilizer application, and improper disposal of animal manure [2]. Data indicated varying degrees of Cd contamination in approximately 7% of China’s arable land [3], while aggravated soil acidification further exacerbated Cd accumulation in crops and livestock, negatively impacting food quality and human health [4]. Given the strong Cd bioaccumulation capacity of rice, China’s national food safety standard restricts Cd to 0.2 mg/kg in grains. Therefore, elucidating the Cd translocation mechanisms in the soil–rice system and devising effective agronomic measures (e.g., straw biochar incorporation) to limit Cd accumulation in rice grains are essential for ensuring food safety and public health.

Straw incorporation is widely adopted to improve soil fertility, carbon sequestration and soil physicochemical properties in sustainable agriculture. However, a consensus has yet to be reached on its effects on soil Cd behavior. Studies reported that straw incorporation could facilitate Cd immobilization by forming complexes with decomposition products, thereby reducing its bioavailability [5]. Additionally, organic fertilizer and straw biochar could elevate soil pH and cation exchange capacity (CEC), further promoting Cd immobilization [6,7,8]. In contrast, recent studies suggested that straw incorporation may potentially increase the availability of metals in soil. Straw decomposition substantially increases soil dissolved organic carbon (DOC), which can form soluble Cd–DOC complexes and promote the mobility and phytoavailability of Cd in paddy soils [9]. Moreover, straw return may aggravate soil acidification, and crop straw from Cd-contaminated fields could even act as a secondary Cd pollution source [10,11].

Notably, most existing studies focused only on single straw management mode or short-term pot experiments, while field-scale investigations on the interactive effects between straw incorporation practices and rice varieties with varying Cd accumulation characteristics remained scarce. This knowledge gap significantly hinders the precise application of straw incorporation in contaminated farmlands. The novelty of this study lies in its field-scale and multi-factor experimental design. We selected two farmlands with distinct Cd concentrations and conducted field experiments based on rice–wheat rotation systems using two rice varieties with high and low Cd accumulation capacities. Against this background, we carried out this field experiment to fill the above research deficiencies.

Nevertheless, under rice–wheat rotation systems, the mechanisms underlying the regulatory effects of straw incorporation on Cd bioavailability, microbial communities, and rice Cd accumulation in Cd-contaminated fields remained unclear. Furthermore, as an essential component of soil microorganisms, filamentous fungi possess strong heavy metal tolerance and potentially contribute to soil cadmium immobilization. Previous studies have verified that native strains such as *Paecilomyces lilacinus* and *Penicillium simplicissimum* could withstand high cadmium and lead stress, suggesting a potential role in Cd immobilization in contaminated fields [12]. To bridge these gaps, five in situ wheat straw incorporation treatments were designed, namely, (1) straw removal (CK, control), (2) straw mulching (SM), (3) straw incorporation (SI), (4) straw incorporation with organic fertilizer (SOI), and (5) straw-derived biochar incorporation (SBI). We hypothesized that modified straw incorporation treatments (SOI/SBI) can potentially immobilize Cd through physicochemical and biological pathways in synergy, ultimately reducing Cd accumulation in rice grains. Based on this research framework, this study aimed to (1) elucidate the effects of different straw incorporation methods on soil physicochemical properties, Cd fraction transformation, and bioavailability in paddy soils; (2) analyze the changes in rhizospheric soil microbial community structure induced by straw incorporation; and (3) determine the impacts of straw incorporation on the Cd accumulation in rice and rice yield. This systematic field comparison of various straw incorporation methods provides valuable insights into the mechanisms governing Cd behavior in soil under different straw management approaches. The findings are expected to offer optimized strategies for straw utilization in rice–wheat rotation systems within Cd-contaminated regions like the Sichuan Basin, while establishing both theoretical foundations and practical references for rice grain Cd risk assessment and mitigation technologies.

## 2. Materials and Methods

### 2.1. Field Experiment Sites

The field experiments were conducted at two sites with Cd-contaminated paddy fields in Sichuan Province, China, i.e., Qiquan Town, Chongzhou City (30°33′ N, 103°38′ E) and Jinxing Village, Mianzhu City (31°34′ N, 104°22′ E). Both sites are characterized by sandy loam soil. Within each site, 700 m^2^ of experimental fields with contrasting soil Cd levels were selected (baseline physicochemical properties in Table S1). Agricultural practices at both sites followed a rice–wheat rotation system, with rice cultivated after wheat harvest. Two hybrid indica cultivars with different Cd accumulation capacities were selected: a low-Cd-accumulating cultivar, Zhenliangyou 8612 (ZLY8612), Anhui Longping Hi-Tech (Xinqiao) Seed Industry Co., Ltd., Hefei, China; and a relatively high-Cd-accumulating cultivar, Yixiangyou 2115 (YXY2115), Sichuan Lvdan Seed Co., Ltd., Chengdu, China.

### 2.2. Experimental Design

The field experiment was conducted simultaneously during the 2024 rice-growing season in Chongzhou (CZ) and Mianzhu (MZ) to evaluate different wheat straw incorporation methods. The experiment employed a split-plot design with the two rice cultivars (ZLY8612, a low-Cd accumulator, and YXY2115, a high-Cd accumulator) as main plots and five straw management practices as sub-plots (CK/control, SM, SI, SOI, and SBI), totaling 10 treatments. Each treatment was arranged in a randomized block design with three replications, totaling 30 plots (3 m × 5 m each). Rice seedlings were manually transplanted at a hill spacing of 16.7 cm and a row spacing of 33.3 cm, corresponding to a planting density of 1.8 × 10^5^ plants per hectare. Detailed fertilization regimes and routine field management practices are provided in the Supplementary Information.

Across all straw incorporation treatments, wheat straw from the preceding cropping season was utilized (in situ incorporation). Following wheat harvest, the straw was chopped into segments of 5 to 10 cm, weighed, and applied at 6000 kg/ha during the rice season. (1) For the CK plots, all wheat straw was manually removed. (2) For the SM plots, the straw was evenly spread on the soil surface. (3) For the SI plots, the straw was incorporated into the 0 to 20 cm soil layer using iron rakes. (4) For the SOI plots, the straw and ALGAE CARBON SINK organic fertilizer (7500 kg/ha, Registration: Sunongfei (2020) Zhunzi 0035; Yancheng Lenong Environmental Technology Co., Ltd., Yancheng, China) were incorporated into the 0 to 20 cm soil layer. (5) For the SBI plots, biochar produced through straw pyrolysis at 500 °C for 2 h was incorporated into the 0 to 20 cm soil layer using iron rakes, with an application rate of 2100 kg/ha (equivalent to the straw input in other treatments). The wheat straw properties for both sites are presented in Table S2.

### 2.3. Sample Collection

During the rice growing season (jointing, heading, and maturity stages), three representative plants were collected from each plot at both experimental sites and pooled to form a composite sample. Concurrently, rhizosphere soil samples (0 to 20 cm depth) were collected using a soil auger from five random locations in each plot, which were homogenized into a single composite sample. Plant samples were separated into roots, stems, leaves, and panicles, thoroughly cleaned, and then oven-dried (105 °C for 30 min for enzyme inactivation, followed by 75 °C to constant weight). The dried samples were weighed, ground, and sieved through a 100-mesh screen for subsequent analysis. One portion of each soil sample was stored at −80 °C for microbial analysis, while the other was air-dried, ground, and sequentially passed through 10-mesh and 100-mesh nylon sieves for physicochemical characterization. Rice yield was measured at maturity for all plots across both sites [13].

### 2.4. Analysis Methods

Soil pH was measured using samples at a solid-to-liquid ratio of 1:2.5 with a pH meter (PHS-3E, Shanghai INESA Scientific Instrument Co., Ltd., Shanghai, China). Soil organic carbon (SOC) content was determined via the potassium dichromate oxidation-volumetric method with external heating [14]. CEC was analyzed using the hexaamminecobalt(III) chloride extraction-spectrophotometric method [15]. DOC was extracted with ultrapure water and quantified using a TOC-5500 analyzer [16]. The soil total Cd content was extracted via microwave digestion with HNO_3_-HF [17]. The DTPA-Cd was measured to assess the bioavailable Cd content [17]. Cd fractions were determined using the BCR sequential extraction procedure [18]. The Cd contents of plant tissues were analyzed after microwave digestion with HNO_3_-H_2_O_2_ [19], followed by quantification using an atomic absorption spectrophotometer (TAS-990, Persee Instruments Co., Ltd., Beijing, China). The detailed digestion procedures for Cd determination in soil and rice tissues are described in Table S3 and Table S4 in the Supplementary Information, respectively. Soil bacterial diversity was analyzed by Shanghai Meiji Biopharmaceutical Technology Co., Ltd. (Shanghai, China) through PCR amplification of 16S rRNA gene V3-V4 regions with primers 338F (5′-ACTCCTACGGGAGGCAGCAG-3′) and 806R (5′-GGACTACHVGGGTWTCTAAT-3′). The analysis methods are detailed in the Supplementary Information [20].

### 2.5. Data Analysis and Statistics

Statistical analysis and data visualization were performed in Microsoft Office 2021, SPSS 25.0, and GraphPad Prism 9.5. Experimental data are presented as mean ± standard deviation (*n* = 3). One-way analysis of variance (ANOVA) with Duncan’s multiple range test was employed to assess intergroup differences, with statistical differences considered significant at *p* < 0.05. For microbial community analysis, α-diversity was calculated based on operational taxonomic units (OTUs), while β-diversity was evaluated using non-metric multidimensional scaling (NMDS) based on Bray–Curtis distances. Redundancy analysis (RDA) was conducted to assess the potential influence of environmental factors on community structure. These microbiome analyses were performed on the Majorbio Cloud Platform (https://www.majorbio.com). The Mantel tests and radial bar chart were implemented in R (v 4.5.1).

## 3. Results

### 3.1. Cd Contents of Various Rice Tissues

**Figure 1:**
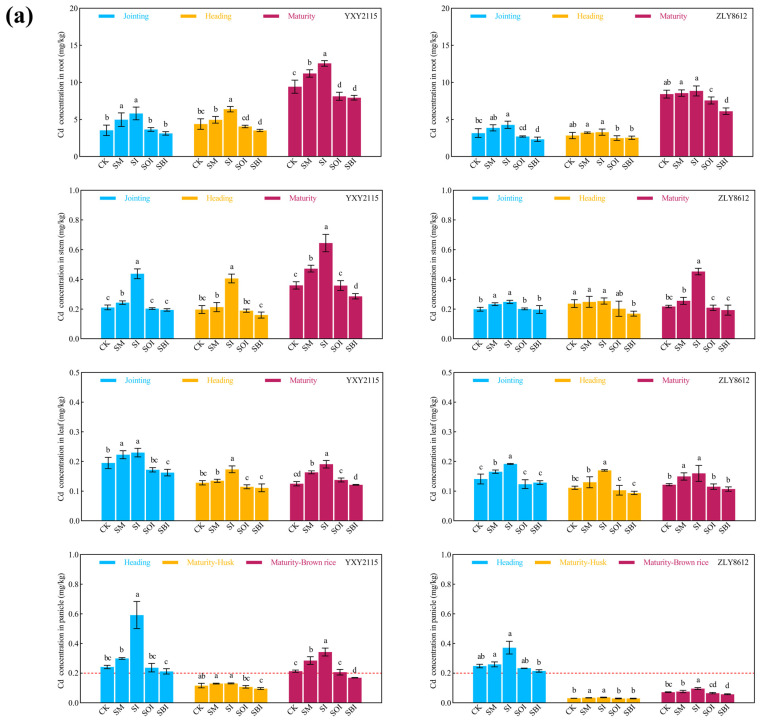

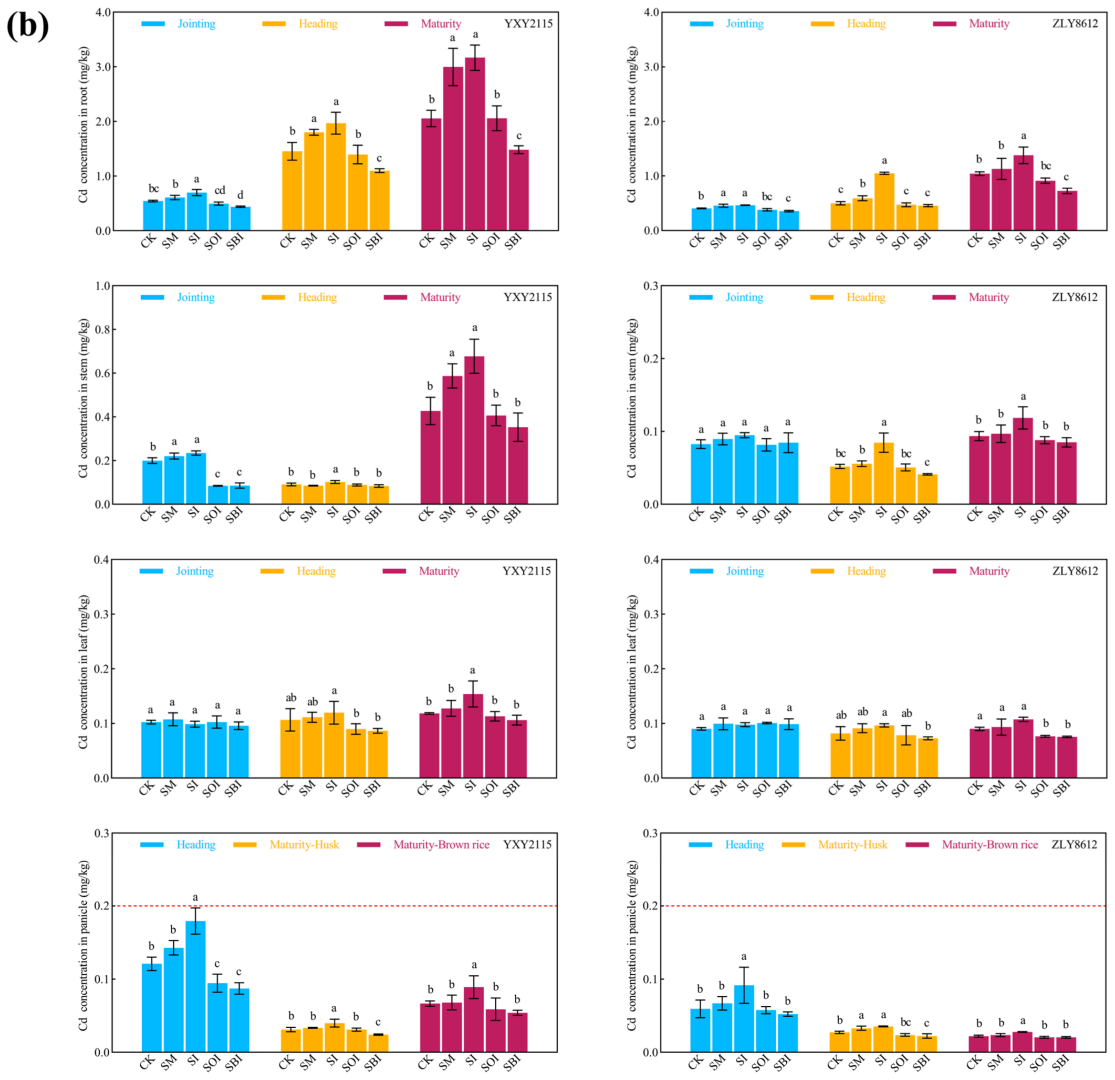
Effects of different straw incorporation methods on Cd accumulation in rice tissues. (**a**) and (**b**) represent Cd-contaminated paddy fields at the CZ and MZ sites, respectively. CK: straw removal; SM: straw mulching; SI: straw incorporation; SOI: straw incorporation with organic fertilizer; SBI: straw-derived biochar incorporation. YXY2115: high-Cd-accumulating rice cultivar; ZLY8612: low-Cd-accumulating rice cultivar. The red dashed line indicates the maximum Cd threshold (0.2 mg/kg) under the Chinese National Food Safety Standard (GB 2762-2022) [21]. Different lowercaseletters indicate statistically significant differences among treatments (*p* < 0.05), and the same significance levels are adopted below.

Systematic analysis of different straw incorporation methods revealed their distinct impacts on Cd accumulation in rice tissues (Figure 1). Consistent variation trends were observed across MZ and CZ sites. The SM and SI treatments increased the Cd concentrations of plant tissues compared to CK. In contrast, the SOI and SBI treatments demonstrated Cd reduction effects, with more pronounced responses observed in YXY2115. Rice tissues from the CZ site consistently exhibited higher Cd concentrations than those from the MZ site, and the cultivar ZLY8612 showed lower Cd levels than YXY2115. The brown rice Cd accumulation was particularly notable. At the CZ site, the SM treatment increased the Cd concentrations in YXY2115 and ZLY8612 by 33.49% (*p* < 0.05) and 5.83%, respectively, compared to CK, while the SI treatment caused more significant increases of 60.34% and 33.98%. At the MZ site, only the SI treatment elevated brown rice Cd in both cultivars (33.83% and 24.89%, *p* < 0.05) relative to CK. Compared to the SI treatment, SOI at CZ significantly reduced brown rice Cd by 39.67% in YXY2115 and 32.09% in ZLY8612, and SBI led to even greater reductions (50.44% and 39.65%, *p* < 0.05). Similar trends were observed at MZ, with significant reductions of 34.04% and 25.36% for SOI and 39.44% and 25.72% for SBI. These results demonstrated the superior mitigation effect of the SBI treatment. Importantly, brown rice Cd concentrations in ZLY8612 at CZ and both cultivars at MZ remained below the food safety threshold (0.2 mg/kg), while only the SBI treatment reduced YXY2115 Cd levels below the threshold at the CZ site.

### 3.2. Soil Cd and DTPA-Cd Contents

**Figure 2:**
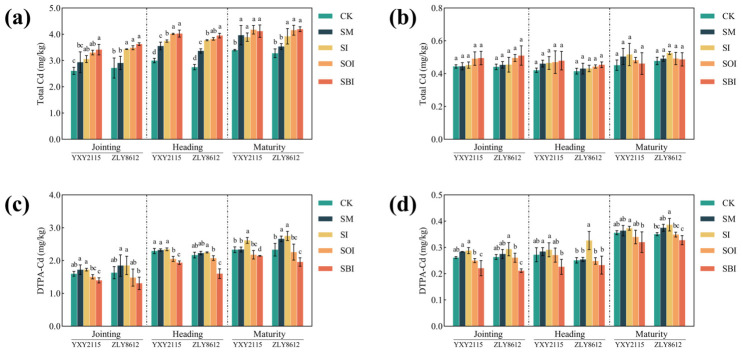
Effects of different straw incorporation methods on total Cd and DTPA-Cd in rhizosphere soil. (**a**,**c**) High-Cd-contaminated paddy field (CZ); (**b**,**d**) Low-Cd-contaminated paddy field (MZ). Different lowercase letters indicate statistically significant differences among treatments (*p* < 0.05).

At the CZ site, all straw treatments increased total soil Cd content, with increases ranging from 9.84% to 38.96% compared to the CK. In contrast, straw incorporation treatments at the MZ site showed no significant effects on total soil Cd content compared to CK (Figure 2). Compared to CK, the SI treatment significantly increased DTPA-Cd levels by an average of 15.03% only at the maturity stage in both rice cultivars at the CZ site. In contrast, compared to the SI treatment, DTPA-Cd levels demonstrated significant decreasing trends under the SOI and SBI treatments. At the CZ site, the reductions averaged 16.36%, 10.06%, and 17.27% under SOI, and 24.22%, 23.09%, and 23.40% under SBI during the three growth stages, respectively. The corresponding average reductions at the MZ site were 12.17%, 15.39%, and 9.37% under SOI, and 25.60%, 25.44%, and 14.67% under SBI. Overall, the DTPA-Cd concentrations in the paddy soil exhibited an increasing trend with the progression of growth stages, peaking at the maturity stage.

### 3.3. Soil SOM, DOC, and CEC Contents

**Figure 3:**
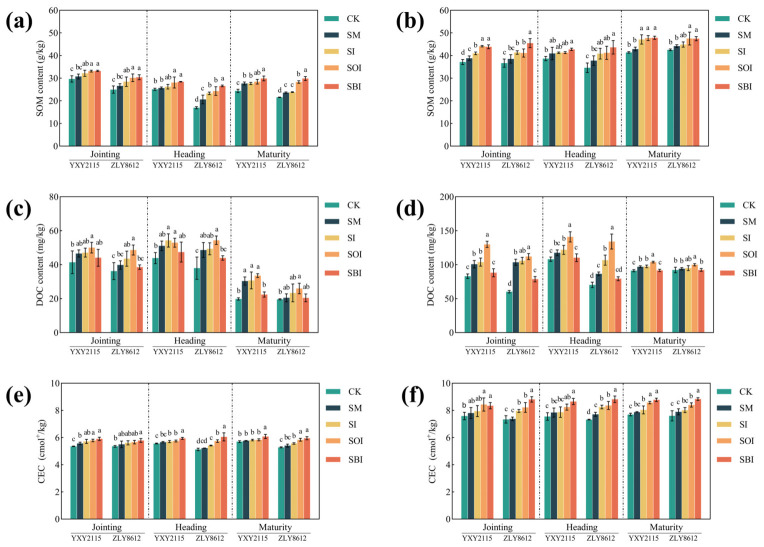
Effects of different straw incorporation methods on rhizosphere soil organic matter (SOM), dissolved organic carbon (DOC), and cation exchange capacity (CEC). (**a**,**c**,**e**) High-Cd-contaminated paddy field (CZ); (**b**,**d**,**f**) Low-Cd-contaminated paddy field (MZ). Different lowercase letters indicate statistically significant differences among treatments (*p* < 0.05).

All straw incorporation methods enhanced soil organic matter (SOM) contents at both CZ and MZ sites (Figure 3), with SOI and SBI treatments showing the most pronounced effects. At the CZ site, SOI significantly increased the SOM content by 16.25–27.69% compared to CK across the three growth stages, while SBI resulted in significant increases of 16.87–30.50%. At the MZ site, SOI significantly elevated SOM levels by 12.95–15.42%, whereas SBI led to significant increases of 13.62–20.97% during the corresponding stages. The DOC contents across treatments ranked as CK < SBI < SM < SI < SOI, with characteristic dynamics of initial increases followed by decreases across rice growth stages. Compared to CK, the DOC content at the CZ site increased by 16.99–37.01% under SI and 27.51–50.91% under SOI across the three growth stages. At the MZ site, DOC increased under SI by 4.91–50.43% and under SOI by 11.12–71.19% across the three growth stages. The results also revealed that at the CZ site, the SBI treatment significantly increased CEC by 9.18–12.43% compared to CK, and at the MZ site, CEC increased by 15.08–17.53% under SBI treatment.

### 3.4. Soil pH and Cd Fractions

**Figure 4:**
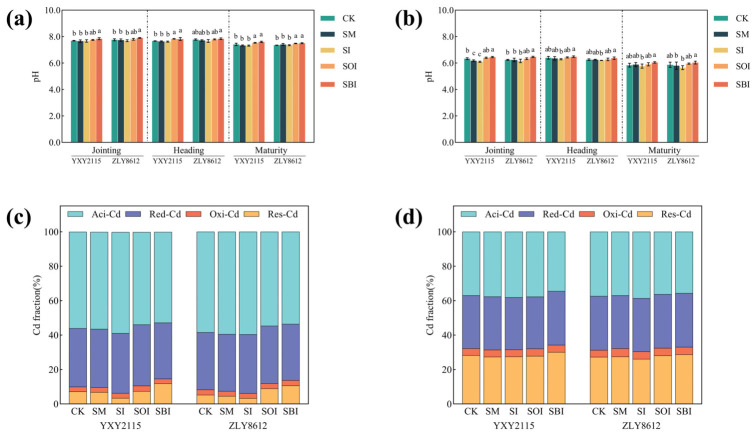
Effects of different straw incorporation methods on pH and cadmium fractions in rhizosphere soil. (**a**,**c**) High-Cd-contaminated paddy field (CZ); (**b**,**d**) Low-Cd-contaminated paddy field (MZ). Aci-Cd: acid-extractable Cd; Red-Cd: reducible Cd; Oxi-Cd: oxidizable Cd; Res-Cd: residual Cd. Different lowercase letters indicate statistically significant differences among treatments (*p* < 0.05).

It was observed that compared to CK, the SOI and SBI treatments tended to increase soil pH (Figure 4), with significant average increases of 0.13 and 0.17 during the maturation period at the CZ site, respectively. Compared to the SI treatment, the SBI treatment increased soil pH by an average of 0.19, 0.18, and 0.22 at CZ and 0.17, 0.10, and 0.18 at MZ across the three growth stages. With alkaline soil conditions at CZ and acidic conditions at MZ, the SM and SI treatments led to a slight trend of soil acidification. Various straw incorporation methods also influenced the distribution of Cd fractions in soil (Figure 4). At maturity, the predominant Cd fractions in rhizosphere soil at the CZ site were acid-extractable Cd (Aci-Cd) and reducible Cd (Red-Cd), whereas those at the MZ site were mainly distributed among Aci-Cd, Red-Cd, and residual Cd (Res-Cd). Compared to CK, the SM and SI treatments increased the proportion of Aci-Cd by an average of 2.52% and 5.79% at the CZ site and by 0.35% and 3.17% at the MZ site, respectively. Conversely, the SOI and SBI treatments decreased the Aci-Cd fraction by 0.54% and 2.92% at CZ and 0.48% and 5.72% at MZ. Compared to the SI treatment, both SOI and SBI increased the proportion of Res-Cd at both sites, with average increases of 13.95% and 21.89% at CZ and 4.54% and 9.76% at MZ, respectively. The SBI treatment showed the most pronounced effect. These findings suggest that SOI and SBI treatments likely reduced Cd bioavailability and environmental risk by facilitating the transformation of Cd from mobile Aci-Cd to more stable Res-Cd.

### 3.5. The Rice Yield and Yield Components

**Figure 5:**
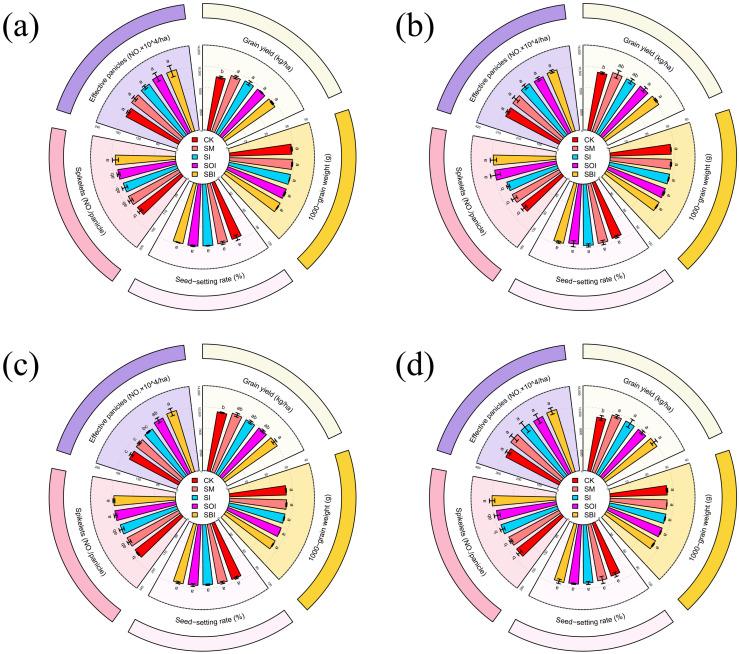
Effects of different straw incorporation methods on yield and yield components of hybrid indica rice, as shown in the radial bar chart. (**a**,**b**) High-Cd-accumulating cultivar YXY2115; (**c**,**d**) Low-Cd-accumulating cultivar ZLY8612; (**a**,**c**) High-Cd-contaminated paddy field (CZ); (**b**,**d**) Low-Cd-contaminated paddy field (MZ). Different lowercase letters indicate statistically significant differences among treatments (*p* < 0.05).

Compared to CK, all straw incorporation treatments increased the grain yield of both rice cultivars (9.20% to 12.43% and 5.89% to 10.68% at CZ/MZ sites with high-Cd cultivar; 4.09% to 6.59% and 11.19% to 15.24% with low-Cd cultivar), with SBI demonstrating the most pronounced yield-enhancing effects. Yield component analysis revealed that the yield improvement primarily originated from increased spikelet numbers per panicle (Figure 5). The SBI treatment increased spikelet numbers per panicle by 8.72% and 23.34% for YXY2115 at the CZ and MZ sites, respectively, while the corresponding increases for ZLY8612 reached 5.70% and 12.19%. Notably, ZLY8612 exhibited superior grain yield performance to YXY2115, with average yield advantages of 5.39% at the CZ site and 23.05% at the MZ site.

### 3.6. Diversity and Structure of Soil Microbial Communities

#### 3.6.1. Diversity of Soil Microbial Communities

Table S5 presents the impacts of different straw incorporation methods on soil microbial α-diversity. In the YXY2115 plots, the ACE, Chao, and Shannon indices under SI, SOI, and SBI treatments were significantly higher than those of CK. In the ZLY8612 plots, the Shannon indices under the SOI and SBI treatments were significantly elevated compared to CK. The β-diversity of microbial communities, assessed via NMDS (stress value < 0.2) based on OTU classification (Figure S1), revealed distinct clustering patterns. In the YXY2115 plots, SBI formed a unique cluster, distinct from other treatments. In the ZLY8612 plots, the SM and SI treatments partially diverged from CK, whereas the SOI and SBI treatments exhibited clear separation.

#### 3.6.2. Structure of Soil Microbial Communities

**Figure 6:**
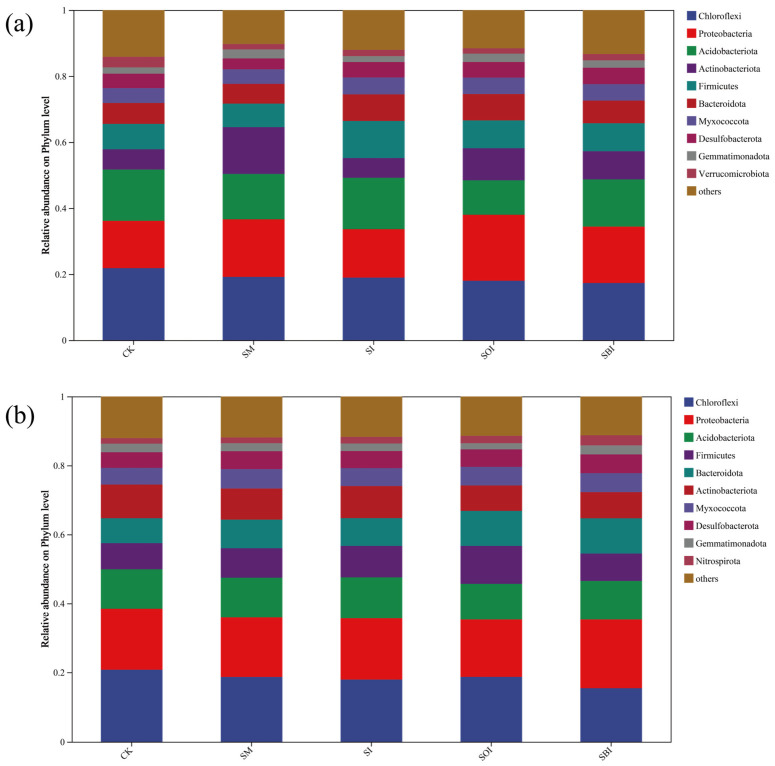
Distribution of dominant microbial phyla under different straw incorporation treatments at the CZ site. (**a**) High-Cd-accumulating cultivar YXY2115; (**b**) Low-Cd-accumulating cultivar ZLY8612.

It was observed that *Chloroflexi, Proteobacteria*, and *Acidobacteriota* were identified as the three dominant phyla at the phylum level (Figure 6). In the YXY2115 plots, the dominant phyla constituted 17.34% to 21.83%, 14.33% to 19.99%, and 10.44% to 15.55%, respectively. Compared to CK, all straw incorporation treatments reduced the relative abundance of *Chloroflexi*. However, SBI and SOI elevated *Proteobacteria* while reducing the abundance of *Acidobacteriota* compared to SI. Similar to YXY2115, in the ZLY8612 plots, these phyla accounted for 15.44% to 20.75%, 17.16% to 19.80%, and 10.43% to 11.97%, respectively. Additionally, SOI led to a higher relative abundance of *Firmicutes*.

**Figure 7:**
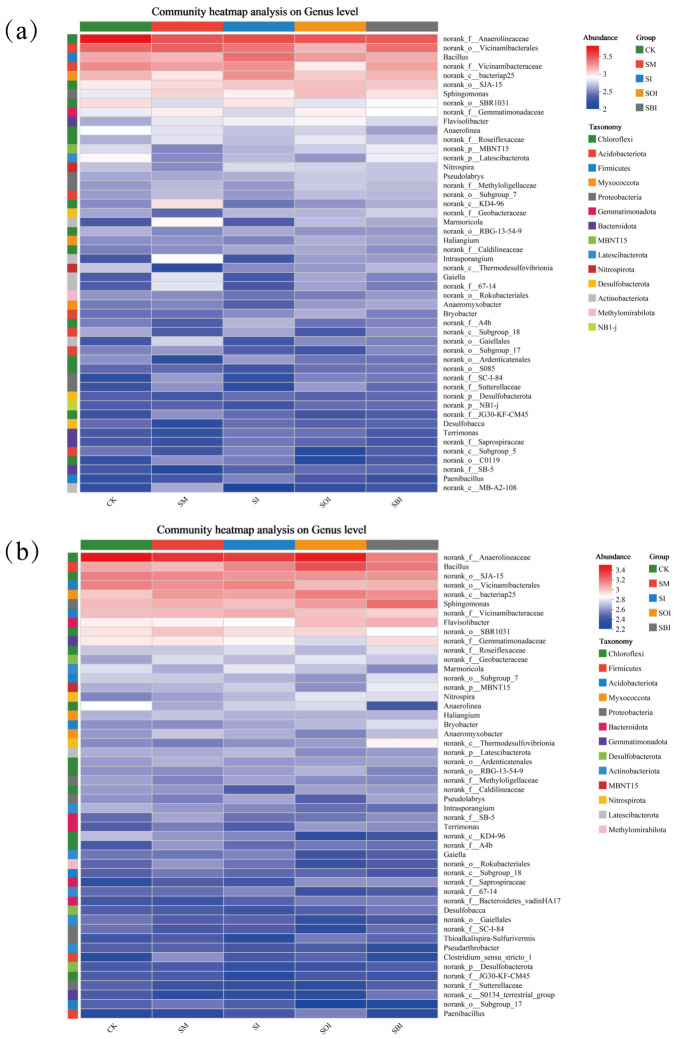
Distribution of dominant microbial genera under different straw incorporation methods at the CZ site. (**a**) High-Cd-accumulating cultivar YXY2115; (**b**) Low-Cd-accumulating cultivar ZLY8612.

Genus-level abundance was also analyzed (Figure 7). In the YXY2115 plots, compared to CK, the SI and SOI treatments enhanced the relative abundance of *Bacillus* (SI: 5.32%; SOI: 1.28%). All straw incorporation treatments (SM, SI, SOI, and SBI) increased the relative abundance of *Sphingomonas* (by 6.16%, 3.38%, 8.02%, and 4.48%, respectively) and *Flavisolibacter* (by 6.14%, 7.09%, 7.76%, and 4.81%, respectively) while decreasing *Anaerolinea* (by 2.29%, 5.19%, 3.96%, and 8.62%, respectively). Notably, the SOI and SBI treatments also promoted functional genera like *Nitrospira*, *Pseudolabrys*, *Marmoricola*, *Bryobacter*, and *Terrimonans*.

Similar trends were observed in the ZLY8612 plots; the SOI and SBI treatments increased *Bacillus* (SOI: 7.02%; SBI: 3.58%), *Sphingomonas* (SOI: 2.53%; SBI: 6.02%), and *Flavisolibacter* (SOI: 3.92%; SBI: 4.82%) while reducing *Anaerolinea* (SOI: 3.00%; SBI: 14.55%) compared with CK. Additionally, functional genera such as *Haliangium* also showed increased relative abundance under SOI and SBI treatments.

#### 3.6.3. Correlations of Soil Microbe Composition and Distribution with Soil Physicochemical Properties

Redundancy analysis (RDA) revealed close correlations between soil microbial community composition and soil physicochemical properties (Figure S2). In the YXY2115 plots, the explained variances of axis1 and axis2 were 28.18% and 15.03%, respectively. DTPA-Cd was negatively correlated with DOC, total-Cd, pH, and SOM, and DTPA-Cd and pH showed a higher explanatory power for bacterial community composition. The ZLY8612 plots displayed a similar association pattern, with RDA axes 1 and 2 accounting for 31.12% and 11.05% of the variance, respectively. Soil DTPA-Cd showed negative correlations with other environmental factors, and DOC, total-Cd, pH, SOM, and CEC exhibited positive correlations. Among these factors, DTPA-Cd and SOM demonstrated a higher explanatory power for the variations in bacterial community than other parameters. This observed divergence likely stemmed from the differential regulation by the root exudates of the two rice cultivars.

### 3.7. Correlation Analysis of Rice Yield Components and Soil Properties

**Figure 8:**
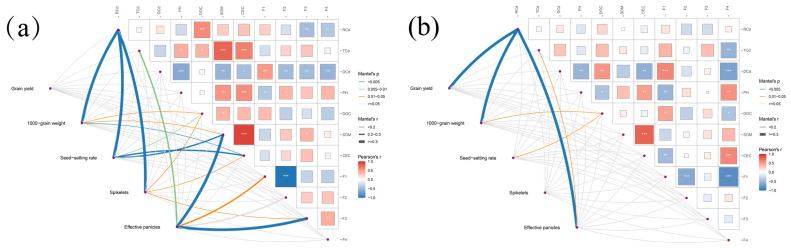
Mantel tests depict the association of yield components and environmental factors. (**a**) High-Cd-contaminated paddy field (CZ); (**b**) Low-Cd-contaminated paddy field (MZ). Significance levels: * *p* < 0.05, ** *p* < 0.01, *** *p* < 0.001. Analyzed parameters: TCd: Total Cd; DCd: DTPA- Cd; F1: Aci-Cd; F2: Red-Cd; F3: Oxi-Cd; F4: Res-Cd; SOM: soil organic matter; DOC: dissolved organic carbon; CEC: cation exchange capacity; pH: soil pH; RCd: Brown rice Cd.

Mantel test results revealed distinct associations between rice yield components and environmental factors across the two study sites (Figure 8). In the CZ region, the relationships were relatively complex: 1000-grain weight, seed-setting rate, Spikelets, and effective panicles were significantly correlated with multiple soil physicochemical factors, while grain yield showed no significant correlation with any environmental factors. In contrast, the associations in the MZ site were simpler, with only a few yield components significantly correlated with a limited number of environmental factors.

Furthermore, the relationships between soil Cd fractions, physicochemical properties, and RCd accumulation were generally consistent across both study regions. Overall, RCd was positively correlated with DCd, DOC, and F1, but significantly negatively correlated with pH. In the Chongzhou area, RCd was also significantly negatively correlated with F3 and F4, suggesting that a higher proportion of stable Cd fractions could reduce Cd uptake. Moreover, DCd was significantly negatively correlated with pH, CEC, and F4, but positively correlated with F1, indicating that soil-available Cd is closely associated with labile Cd fractions. Meanwhile, pH showed a significant negative correlation with F1 and a positive correlation with F4, further suggesting that increased soil pH promotes the transformation of Cd into more stable fractions and enhances Cd retention capacity.

Collectively, soil physicochemical properties were closely linked to the distribution and bioavailability of Cd fractions, influencing Cd accumulation in rice grains. Additionally, these properties played a crucial role in shaping key yield components, thereby impacting rice yield formation.

## 4. Discussion

### 4.1. Effects of Straw Incorporation Methods on Cd Availability and Soil Physicochemical Properties in Paddy Fields

Previous studies have demonstrated that the Cd accumulated in straw can be released during decomposition, thereby increasing Cd contamination risks [22]. The present findings are generally consistent with these previous observations. This research suggested that soil Cd accumulation levels may be closely associated with both the Cd content of wheat straw and regional Cd background values in the soil. In high-Cd regions, the elevated Cd content of wheat straw from the previous season led to increased total soil Cd following straw incorporation. In low-Cd regions, straw incorporation showed minimal effects on total soil Cd, likely due to lower Cd content in the straw. DTPA-Cd represents the phytoavailable fraction of Cd in soil systems. The SM and SI treatments increased the DTPA-Cd content, possibly due to Cd release from straw and Cd activation under decreased soil pH, consistent with previous reports of enhanced Cd mobility under straw incorporation in double-cropping systems [23]. Conversely, the SOI and SBI treatments appeared to reduce DTPA-Cd, possibly through the following mechanisms: (1) The organic matter complexation through oxygen-containing functional groups (carboxyl, phenolic, and hydroxyl) effectively immobilized Cd by forming stable chelates [24]. (2) Elevated soil pH likely suppressed Cd mobility, consistent with the established negative relationship between pH and Cd activity [25]. (3) Biochar may induce Cd immobilization through electrostatic adsorption, ion exchange, co-precipitation with Fe/Mn (hydr)oxides, and coordination with organic ligands [26]. Notably, the observed DTPA-Cd increases during late growth stages correlated with field drainage-induced oxidative conditions. This trend might be explained by drainage-induced soil acidification and consequent transformation of Fe/Mn (hydr)oxide-bound Cd into exchangeable fractions, potentially enhancing Cd phytoavailability [27]. Soil Cd fractions determine Cd bioavailability and environmental risks, with rice Cd accumulation predominantly governed by non-residual fractions, particularly Aci-Cd being the most readily phytoavailable form in paddy fields [28,29]. Both SM and SI increased the proportion of Aci-Cd, whereas SOI and SBI reduced the proportion of Aci-Cd while promoting Cd transformation into the more stable Res-Cd fractions. This divergence might be driven by organic fertilizer-mediated Cd redistribution via DOM, where humic substances may facilitate the conversion of acid-extractable Cd to reducible, oxidizable and residual forms [30]. Biochar likely immobilized Cd through its high alkalinity, mineral content, and functional groups [31]. Meanwhile, biochar altered soil conditions to promote Cd complexation and the formation of carbonate or phosphate precipitates [32]. Collectively, these observations suggest that optimized straw incorporation strategies may facilitate Cd stabilization and reduce rice Cd accumulation.

### 4.2. Mechanisms of Cd Bioavailability Regulation Through Synergistic Soil Factors Under Straw Incorporation Practices

Soil pH is a crucial factor controlling Cd availability. Higher pH levels may promote the formation of insoluble Cd compounds (e.g., Cd(OH)_2_ or CdCO_3_) [33], whereas lower pH levels increase Cd solubility and mobility. The SOI and SBI treatments increased soil pH and reduced Cd activity, which may contribute to Cd accumulation in rice tissues (Figure 1). Notably, SBI led to the most pronounced pH elevation, suggesting its superior Cd immobilization potential to other straw management methods. SOM displayed a strong Cd affinity through adsorption and chelation processes, forming stable organometallic complexes [34]. Although DOC has been reported to enhance Cd mobility by serving as a carrier [22], SOI still significantly reduced both DTPA-Cd and brown rice Cd concentrations. These results align with the previous findings [34], implying that organic fertilizer application enhanced Cd immobilization through pH elevation and organic matter content increments, promoting Cd transformation into less available forms (e.g., organic-bound, Fe oxide-bound, and carbonate-bound fractions) [35]. Our results indicated that the SBI treatment achieved the best performance. This effect was closely related to its remarkable improvement in soil CEC, which is a key indicator of cation exchange and retention capacity and shows a negative correlation with Cd availability [25]. Correlation analysis revealed the negative relationships of DTPA-Cd with pH and CEC, suggesting the synergistic effects of pH-mediated precipitation and organic complexation in Cd stabilization. Collectively, these findings have important implications for field straw management policies: conventional direct straw return (SM/SI) is not suitable for high-Cd paddy fields, while SBI provides a sustainable strategy for safe straw disposal and long-term soil health maintenance in contaminated farmland.

### 4.3. Impact of Differential Straw Incorporation Methods on Soil Microbial Community Structure

Rhizosphere microorganisms constitute a complex community that is widely recognized as the second genome of plants. The collective genetic pool of rhizosphere microbiota is far larger than that of the host plant, and it plays indispensable roles in organic matter transformation, nutrient cycling, and soil–plant interactions [36,37]. Our results indicated that straw incorporation methods could reshape microbial community structures by altering carbon supply patterns, with the SOI and SBI treatments showing particularly pronounced effects on α-diversity and β-diversity, likely resulting from enhanced carbon availability (via straw decomposition products/organic fertilizers) and improved microhabitats (e.g., biochar pore structures), which collectively promoted microbial proliferation and community restructuring [38,39]. At the phylum level, *Chloroflexi, Proteobacteria*, and *Acidobacteriota* emerged as the dominant taxa, consistent with their key roles in organic matter decomposition, soil fertility enhancement, nutrient cycling, and ecosystem stability. Contrary to previous reports [40], we observed a decreased abundance of *Chloroflexi* in soils with straw incorporation treatments. While this phylum typically utilizes exogenous organics (e.g., organic acids or alcohols) to establish proton gradients and generate energy via light-sensitive proteins [41], the high Cd content in straw may inhibit energy-metabolizing enzymes, thus suppressing their activity despite increased organic matter. SBI increased the relative abundance of *Proteobacteria*, which can mitigate Cd toxicity by expressing specific Cd-binding proteins, Cd transporters, and antioxidant enzymes, enabling their survival and proliferation in Cd-contaminated environments [42]. These findings could explain the lower DTPA-Cd levels observed under the SBI treatment. Additionally, *Acidobacteriota* thrived under the SI treatment, likely due to their pH sensitivity and preference for acidic conditions [43]. Furthermore, certain *Firmicutes* with robust antioxidant systems can alleviate reactive oxygen species induced by heavy metals, suppress oxidative stress, and protect cellular integrity [44]. Their proliferation under the SOI treatment, possibly driven by organic fertilizer input, may contribute to the reduced Cd accumulation in ZLY8612 rice tissues. Furthermore, *Bacillus* facilitates straw degradation through cellulase secretion, thereby enhancing SOM [45]. While promoting plant growth, suppressing pathogens, and participating in nitrogen cycling and antioxidant functions, *Sphingomonas* exhibits heavy metal immobilization capacity [46]. Meanwhile, *Flavisolibacter* potentially contributes to organic pollutant degradation in contaminated environments [47]. Conversely, the relative abundance of *Anaerolinea* decreased, which is known for decomposing DOM and regulating soil carbon cycling. Its suppression may slow DOM decomposition, promoting the formation of more stable Cd-DOM complexes and consequently reducing Cd bioavailability [48]. Additionally, *Nitrospira* enrichment could be linked to biochar-enhanced nitrification [49]. *Pseudolabrys* exhibits a link with potential denitrification-related bacterial functions [50], and *Marmoricola* may serve as a sensitive bioindicator for Cd/As [51]. RDA established strong linkages between microbial communities and soil key parameters (DTPA-Cd, SOM, pH), indicating tight soil-microbe coupling. Notably, the observed Cd accumulation differences between cultivars may be closely tied to variations in rhizosphere microbiome, as low-Cd-accumulating cultivars often exhibit greater microbial diversity, potentially through enhanced antioxidant defense and Cd immobilization [52]. In this study, SBI demonstrated superior Cd regulation, likely due to (1) biochar-mediated soil conditioning that boosts bacterial metabolic activity, energy production, and nutrient cycling efficiency, and (2) synergistic reinforcement of bioremediation alongside physical Cd adsorption [53,54]. Furthermore, Filamentous fungi may also contribute to Cd immobilization and tolerance in contaminated soils. Soil microbiota including filamentous fungi play crucial ecological functions in regulating soil nutrient cycling and microbial community interactions [55]. Rhizosphere and endophytic fungal communities are closely shaped by agricultural management and soil environmental conditions [56]. Beneficial soil bacteria can suppress the infection of pathogenic filamentous fungi through direct intercellular contact [57], while indigenous filamentous fungi isolated from paddy soils also display great potential for heavy metal immobilization and soil remediation [58]. Future studies should investigate the contributions of filamentous fungi under field conditions, particularly in rice paddies with straw incorporation.

### 4.4. Effects of Different Straw Incorporation Methods on Cd Accumulation in Rice

This study indicated that different straw incorporation methods may significantly influence Cd accumulation in rice, with more pronounced effects observed in high-Cd soils and high-Cd-accumulating cultivars (YXY2115). The Cd distribution across rice tissues ranked as roots > stems > leaves > husks > brown rice (Figure 1), and DTPA-Cd was positively correlated with brown rice Cd concentration. Notably, the SM and SI treatments using high-Cd straw (2.82 mg/kg, CZ) caused the brown rice Cd concentrations to exceed the national safety threshold (0.2 mg/kg). In contrast, treatments with low-Cd straw (0.59 mg/kg, MZ) led to Cd levels consistently below that threshold, highlighting a close correlation between brown rice Cd accumulation and residual Cd in straw.

Diverging from prior research [40,59], this study innovatively employed the in situ incorporation of Cd-contaminated wheat straw in polluted fields, which better reflected actual agricultural production conditions. While CK minimized Cd accumulation in rice tissues, it resulted in the lowest yields due to soil nutrient depletion. In high-Cd-contaminated paddy fields, SM and SI increased YXY2115 yields but caused the brown rice Cd content to exceed the safety threshold (>0.2 mg/kg), whereas ZLY8612 maintained yield superiority and compliant Cd levels (<0.2 mg/kg). In low-Cd-contaminated paddy fields, SM and SI boosted yields without exceeding the Cd safety threshold in either cultivar, demonstrating the higher safety of straw incorporation under low Cd background conditions. Although SOI improved YXY2115 yields, the brown rice Cd still exceeded the safety threshold at the CZ site. Remarkably, SBI enhanced rice yields at both sites while consistently maintaining grain Cd concentrations below 0.2 mg/kg, demonstrating a unique “yield increase–Cd reduction” synergy. Nevertheless, this study still has certain limitations. This study was conducted only in a single rice growing season at two sites within a limited area, which confines the applicability of the research conclusions. Meanwhile, limitations remain in microbial community analysis. Multi-year and multi-site field experiments combined with metagenomic analysis will be carried out in follow-up studies to further clarify the underlying mechanisms.

In summary, this study demonstrates that in slightly Cd-contaminated regions (represented by the MZ site in this study), cost-effective straw management practices (SM or SI) can be adopted for yield improvement. Conversely, in heavily Cd-contaminated regions (represented by the CZ site in this study), SOI or SBI should be implemented alongside low-Cd-accumulating cultivars to simultaneously ensure productivity and food safety. These findings can provide practical references for formulating targeted straw management policies and sustaining long-term soil health in Cd-polluted rice cultivation areas. Nevertheless, the present study has a certain limitation regarding the biochar application. Due to the lack of direct physicochemical characterization data of the biochar (e.g., proximate analysis, elemental composition, and surface structure), it is difficult to further elaborate the intrinsic mechanism by which biochar regulates the fraction transformation and availability of cadmium in the soil–rice system. Future studies should conduct comprehensive characterization of biochar properties to more clearly reveal the immobilization and stabilization mechanisms of cadmium in cadmium-contaminated paddy fields.

## 5. Conclusions

This field experiment elucidated the effects of different wheat straw incorporation methods on Cd accumulation in subsequent rice crops within a rice–wheat rotation system. The key findings are as follows: (1) The SM and SI treatments tended to lower soil pH and elevated DOC levels, promoting Cd solubilization and enhancing its mobility, thereby increasing brown rice Cd concentrations. In contrast, the SOI and SBI treatments facilitated Cd transformation into residual fractions by increasing SOM, pH, and CEC, effectively reducing grain Cd accumulation. SBI exhibited the most pronounced immobilization effect. (2) SOI and SBI enhanced soil microbial α-diversity and altered β-diversity, characterized by increased relative abundances of *Proteobacteria*, decreased *Chloroflexi* and *Acidobacteriota*, and enrichment of functional genera (*Bacillus, Sphingomonas,* and *Flavisolibacter*), synergistically promoting Cd immobilization. (3) The low-Cd-accumulating cultivar ZLY8612 showed better performance than YXY2115 across all treatments. In heavily Cd-contaminated regions, the low-Cd-accumulating rice cultivar ZLY8612 maintained brown rice Cd below the 0.2 mg/kg safety threshold in all treatments, while the high-Cd-accumulating rice cultivar YXY2115 only met this standard under the SBI treatment. Both cultivars complied with food safety standards in light-Cd-contaminated fields regardless of treatments. Hence, distinct straw management strategies should be adopted based on Cd contamination levels. These findings are applicable to the local study area, and can also offer practical guidance for Cd risk prevention in farmland with similar pollution characteristics. In addition, further research is required to explore the community composition and ecological functions of other soil fungi and microorganisms, as well as their fundamental roles in regulating Cd migration, transformation and accumulation in the soil–rice rotation system.

## Data Availability

The original contributions presented in the study are included in the article/Supplementary Materials, further inquiries can be directed to the corresponding author.

## Supplementary Materials

The following supporting information can be downloaded at: https://www.mdpi.com/article/10.3390/foods15122057/s1, Table S1: Physicochemical properties of the tested soils; Table S2: Characterization of Cd content in tissues of previous wheat; Table S3: Cd digestion procedure in soil; Table S4: Cd digestion procedure in rice tissues; Table S5: Effects of different straw incorporation methods on soil microbial α-diversity (CZ); Figure S1: Effects of different straw incorporation methods on rhizosphere soil microbial β-diversity at the CZ site. (a) High-Cd-accumulating cultivar YXY2115; (b) Low-Cd-accumulating cultivar ZLY8612; Figure S2: Redundancy analysis (RDA) of soil microbial communities under different straw incorporation methods at the CZ site. (a) High-Cd-accumulating cultivar YXY2115; (b) Low-Cd-accumulating cultivar ZLY8612.
