# Supplementary material for: Impacts of In Situ Wheat Straw Incorporation Methods on Cadmium Behavior in Soil–Rice Systems
Source: Foods. 2026 Jun 6;15(12):2057. doi: 10.3390/foods15122057 (PMC13298096; doi:10.3390/foods15122057)
Supplement: Supplementary file 1 [file foods-15-02057-s001.zip › foods-4291265-supplementary.pdf]

Supplementary Information

Impacts of In Situ Wheat Straw Incorporation Methods on Cadmium Behavior in Soil–Rice Systems

Captions:

Section I: Supplementary tables and figures

Section II: Analysis methods

Section III: Field experiment photograph at the Mianzhu site

Section I: Supplementary tables and figures

Table S1

Physicochemical properties of the tested soils.

Sites	pH	CEC cmol/kg	Total-Cd mg/kg	DTPA-Cd mg/kg	SOM g/kg	Available-N mg/kg	Available-P mg/kg	Available-K mg/kg
Chongzhou	7.57	5.54	2.68	1.26	27.75	87.88	16.06	143.26
Mianzhu	6.31	6.36	0.59	0.33	49.84	83.25	25.30	130.75

Table S2

Characterization of Cd content in tissues of previous wheat.

Sites	Wheat variety	Wheat straw mg/kg	Wheat grain mg/kg
Chongzhou	Hanlumai-C	2.82	1.11
Mianzhu	Chuanmai-1247	0.59	0.10

Table S3

Cd digestion procedure in soil.

Step	Temperature/°C	time/min
1	80	2
2	120	2
3	150	2
4	180	5
5	190	30

Table S4

Cd Digestion Procedure in rice tissues.

Step	Temperature/℃	time/min
1	80	2
2	120	2
3	150	2
4	180	5
5	190	20

Table S5

Effects of different straw incorporation methods on soil microbial α-diversity (CZ).

Cultivar	Treatment	Richness index		Diversity index
		ACE	Chao	Shannon
	CK	5778.96±179.90b	5703.93±131.84b	7.20±0.03c
	SM	5716.82±146.48b	5676.75±197.03b	7.24±0.03bc
YXY2115	SI	6341.99±48.05a	6242.10±39.49a	7.26±0.04ab
	SOI	6383.73±93.41a	6236.87±110.98a	7.30±0.02a
	SBI	6320.39±135.65a	6217.14±152.03a	7.29±0.02ab
	CK	6213.20±20.06a	6091.57±76.60a	7.22±0.03b
	SM	6245.65±155.46a	6176.11±153.75a	7.28±0.01ab
ZLY8612	SI	6233.20±112.42a	6143.19±93.98a	7.23±0.05b
	SOI	6319.85±85.15a	6254.04±90.81a	7.31±0.04a
	SBI	6365.15±60.86a	6215.03±66.78a	7.30±0.01a

Note: α-diversity indices of soil microbial communities at the maturity stage.

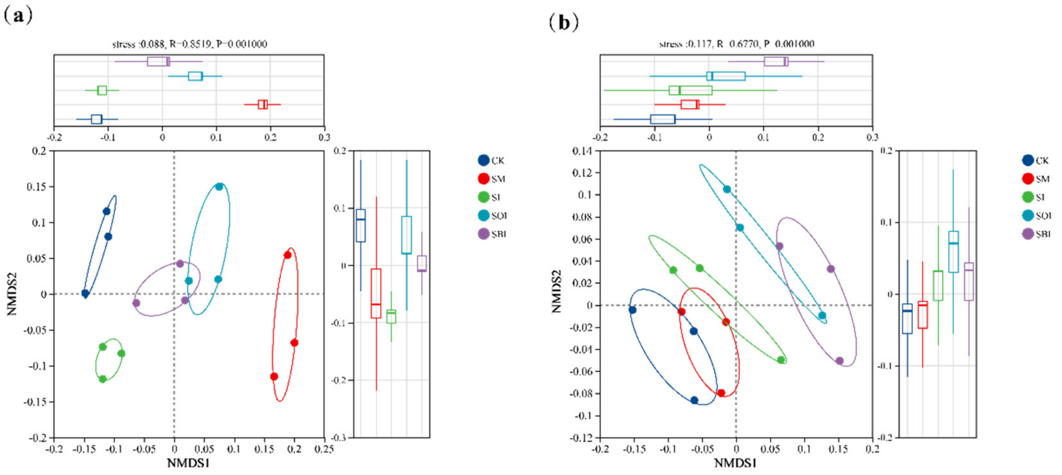

Figure S1. Effects of different straw incorporation methods on rhizosphere soil microbial β-diversity at the CZ site. (a) High-Cd-accumulating cultivar YXY2115; (b) Low-Cd-accumulating cultivar ZLY8612.

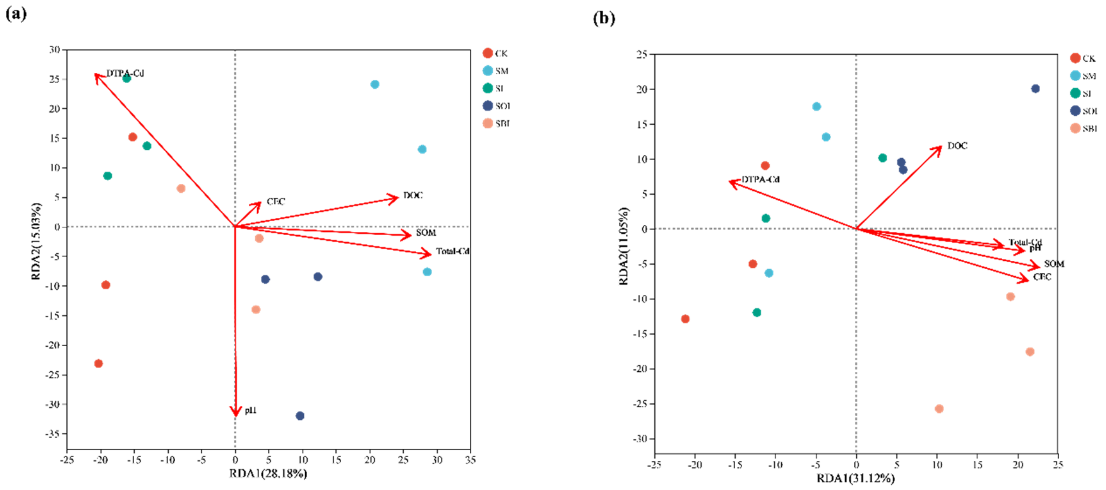

Figure S2. Redundancy analysis (RDA) of soil microbial communities under different straw incorporation methods at the CZ site. (a) High-Cd-accumulating cultivar YXY2115; (b) Low-Cd-accumulating cultivar ZLY8612.

Section II: Analysis methods

Soil pH:

Briefly, 10.0 g of air-dried soil sample (passed through a 2-mm sieve) was weighed and added to a 50 mL beaker. Then, 25 mL of deionized water (soil-to-water ratio 1:2.5) was added, and the mixture was stirred with a glass rod for 2 min to ensure thorough homogenization. The suspension was allowed 30 min to stabilize before measurement. The PHS-3E pH meter was calibrated using standard buffer solutions.

Soil organic matter (SOM)[1]:

Briefly, 0.3 g of soil (100-mesh) was weighed and placed into a 50 mL hard glass tube, and 0.1 g of silver sulfate was added. Subsequently, 5.00 mL of 0.8 mol/L potassium dichromate solution and concentrated sulfuric acid were added. The mixture was then heated in an oil bath at 180°C for 5 min. Afterward, the solution was transferred to a conical flask and washed with deionized water. The analyte was determined by titration using 0.2 mol/L ferrous sulfate solution.

Soil cation exchange capacity (CEC)[2]:

Briefly, 0.5 g of soil (10-mesh, 2 mm) was accurately weighed and placed into a 50 mL centrifuge tube. Then, 10.0 mL of a 1.66 cmol/L hexamminecobalt(III) chloride solution was added. The mixture was then shaken using a thermostatic shaker for 60 min at 25 ± 1°C. After shaking, the sample was centrifuged at 5000 rpm for 10 min. The supernatant was collected, and the absorbance was measured at wavelengths of 475 nm and 380 nm using a spectrophotometer.

Dissolved organic carbon (DOC)[3]:

Briefly, 5.00 g of air-dried soil (100-mesh) was accurately weighed and placed into a 50 mL centrifuge tube. Then, 25 mL of ultrapure water was added, maintaining a soil-to-water ratio of 1:5. The mixture was then shaken at 180 rpm for 30 min. After extraction, the sample was centrifuged at 5000 rpm for 10 min. The supernatant was filtered through a 0.45 μm aqueous membrane. The filtrate was analyzed for total organic carbon (TOC) using a TOC-5500 analyzer (METASH).

Total-Cd in soil [4]:

Briefly, 0.1000 g of the air-dried soil sample after passing through a 100-mesh sieve was weighed and placed in a polytetrafluoroethylene (PTFE) digestion vessel (M3 microwave digestion system). Sequentially, 7 mL of concentrated nitric acid (HNO_3, analytical grade) and 1 mL of hydrofluoric acid (HF, analytical grade) were added. The sample was subjected to microwave digestion using a gradient heating program (Table S3). After cooling, the digestion solution was transferred to a 50 mL volumetric flask and diluted to the mark with ultrapure water. The resulting solution was filtered through a 0.45 μm membrane and analyzed using a TAS-990 atomic absorption spectrophotometer.

DTPA-Cd in soil (DTPA-Cd)[4]:

Briefly, 5.00 g of the air-dried soil sample after passing through a 100-mesh sieve was weighed and placed into a 50 mL centrifuge tube. Then, 25 mL of DTPA extraction solution (a mixture of 0.1 mol/L triethanolamine (TEA), 0.01 mol/L calcium chloride ($CaCl_2$), and 0.005 mol/L diethylenetriaminepentaacetic acid (DTPA), with pH adjusted to 7.3) was added. The mixture was shaken for 120 min for extraction and then centrifuged at 5000 rpm for 10 min. The supernatant was filtered through a 0.45 μm membrane. The filtrate was analyzed using a TAS-990 atomic absorption spectrophotometer.

Soil Cd fraction [5]:

The BCR sequential extraction method was employed to determine the fractions of acid-extractable (Aci-Cd), reducible (Red-Cd), oxidizable (Org-Cd), and residual (Res-Cd) cadmium. Specifically, 0.5 g of soil sample was mixed with different extracting reagents and shaken for 16 h. At the end of each step, the solution and precipitate were separated by centrifugation at 4000 g for 15 min. The supernatant was then filtered through a 0.45 μm filter. The extracted cadmium concentrations were determined using a TAS-990 analyzer.

Acid-extractable Cd (Aci-Cd) was obtained by extracting with 20 mL of 0.11 M acetic acid solution.

Reducible Cd (Red-Cd) was extracted using 20 mL of 0.5 M hydroxylamine hydrochloride solution.

Oxidizable Cd (Org-Cd) was extracted using 10 mL of hydrogen peroxide and 20 mL of 1.0 M ammonium acetate solution.

Residual Cd (Res-Cd) was digested by microwave using 7 mL of concentrated nitric acid (analytical grade) and 1 mL of hydrofluoric acid (analytical grade).

Cd concentrations in different rice tissues [6]：

Briefly, 0.2000 g of rice plant sample (100-mesh) was accurately weighed and placed into a polytetrafluoroethylene (PTFE) digestion vessel (M3 microwave digestion system). Then, 7 mL of concentrated nitric acid (HNO_3, analytical grade) and 1 mL of hydrogen peroxide (H_2O_2, analytical grade) were added sequentially. Microwave digestion was performed using a gradient heating program (Table S4). After cooling, the digestion solution was transferred to a 50 mL volumetric flask and diluted to the mark with ultrapure water. The diluted solution was filtered through a 0.45 μm membrane and analyzed using a TAS-990 atomic absorption spectrophotometer.

Prior to instrumental analysis, the cadmium standard stock solution was serially diluted to obtain a gradient of standard concentrations. The absorbance was measured under optimized instrumental parameters, including a cadmium hollow cathode lamp, wavelength of 228.8 nm, and spectral bandwidth of 0.4 nm. A calibration curve was constructed, and the Cd concentrations in samples were calculated via linear regression analysis. Certified reference material (CRM) GBW08612 (cadmium single-element standard solution) was used for method validation and quality control throughout the analysis. Comprehensive quality assurance and quality control (QA/QC) protocols were strictly followed throughout the entire analytical procedure. The calibration curve yielded a correlation coefficient (R^2 > 0.999), demonstrating excellent linearity. Reagent blanks were prepared and analyzed alongside each digestion batch to subtract background interference derived from reagents and experimental surroundings. All soil samples were processed and determined in three parallel replicates, with the relative standard deviation (RSD) below 5%, indicating good analytical precision. The instrumental and method detection limits were established based on repeated blank

measurements, and all sample Cd concentrations were well above the detection limit. The above QA/QC measures guaranteed reliable accuracy and precision for Cd determination in soil.

Rice yield traits and Fertilization measures [7]:

At maturity, 30 representative rice plants were randomly selected from each experimental plot to survey the number of effective panicles and calculate the average value. Based on the average panicle number, 5 rice plants with consistent growth were randomly selected from each plot for further analysis. The number of empty grains, the number of filled grains, and the thousand-grain weight were measured. At harvest, plants from the border rows were removed, and those in the central area were harvested. The yield per unit area (kg/ha) was then calculated based on the actual number of plants harvested.

Fertilizers were applied uniformly across all treatments. Nitrogen was applied as urea (150 kg/ha) in four split applications at a ratio of 3:3:2:2 for basal, tillering, panicle initiation, and spikelet development stages, respectively. Phosphorus (P_2O_5 90 kg/ha) and potassium (K_2O 150 kg/ha) were applied as basal fertilizers in a single dose. All plots were separated by 40 cm wide and 25 cm high ridges covered with black plastic film to prevent water and nutrient exchanges between treatments. Other field management practices, including pest and disease control, followed standard local protocols for high-yield rice production.

Determination of rhizosphere soil bacteria [8]:

Total DNA of the soil microbial genomes was extracted using the E.Z.N.A.® soil DNA kit (Omega Bio-tek, Norcross, GA, US). A 1% agarose gel electrophoresis was employed to assess the quality of the extracted DNA, and NanoDrop2000 (Thermo Scientific, US) was used to measure DNA concentration and purity. The hypervariable region V3-V4 of the bacterial 16S rRNA gene was amplified with primer pairs 338F (5'-ACTCCTACGGGAGGCAGCAG-3') and 806R(5'-GGACTACHVGGGTWTCTAAT-3') by a T100 Thermal Cycler PCR thermocycler (BIO-RAD, US). The PCR product was extracted from 2% agarose gel and purified using the PCR Clean-Up Kit (YuHua, Shanghai, China) according to the manufacturer's

instructions and then quantified using Qubit 4.0 (Thermo Fisher Scientific, US). Sequencing procedures were performed using the Illumina Nextseq 2000 platform. MiSeq sequencing was provided by Shanghai Meiji Biopharmaceutical Technology Co., Ltd.

Raw sequencing reads were quality-filtered, trimmed, and assembled using Trimmomatic and FLASH software. Low-quality reads, short sequences, and ambiguous bases were removed. Clean sequences were clustered into OTUs at 97% similarity using the Usearch platform. Taxonomic classification was annotated against the Silva database (Release 128) with a confidence threshold of 0.7. To eliminate the influence of uneven sequencing depth, all samples were normalized by rarefaction to the minimum read count before alpha and beta diversity analysis. All bioinformatics analyses were performed on the Majorbio Cloud Platform.

Section III: Field experiment photograph at the Mianzhu site

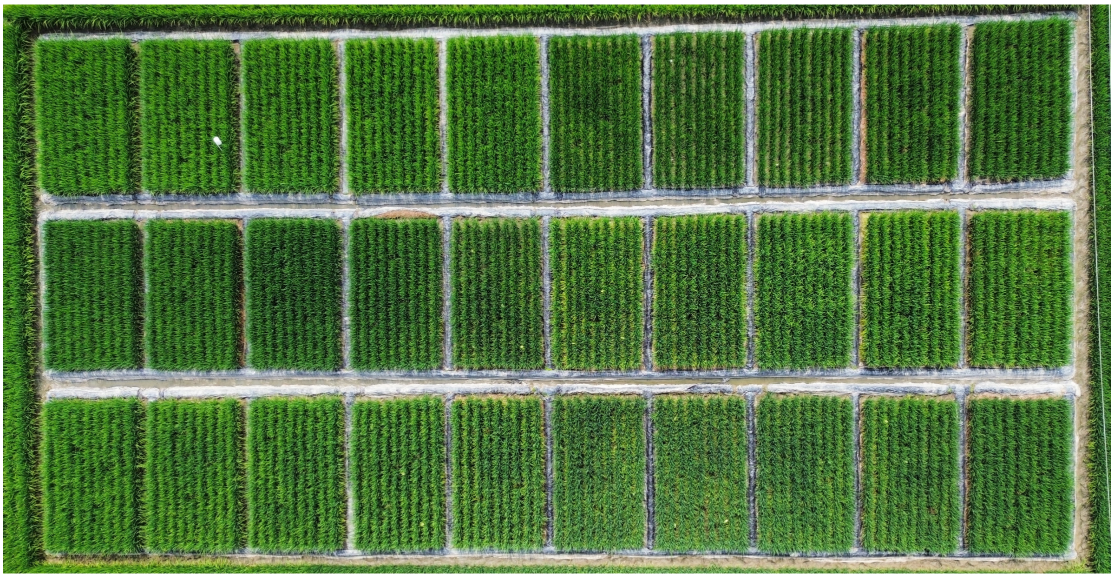

References

1. Nelson, D.W.; Sommers, L.E. Total Carbon, Organic Carbon, and Organic Matter. In *Methods of Soil Analysis*; 1996; pp. 961-1010.
2. Aran, D.; Maul, A.; Masfaraud, J.-F. A spectrophotometric measurement of soil cation exchange capacity based on cobaltihexamine chloride absorbance. *Comptes Rendus Geoscience* **2008**, *340*, 865-871, doi:10.1016/j.crte.2008.07.015.
3. Huang, M.; Zhou, M.; Li, Z.; Ding, X.; Wen, J.; Jin, C.; Wang, L.; Xiao, L.; Chen, J. How do drying-wetting cycles influence availability of heavy metals in sediment? A perspective from DOM molecular composition. *Water Research* **2022**, *220*, 118671, doi:10.1016/j.watres.2022.118671.
4. Huang, Q.; Wang, Y.; Qin, X.; Zhao, L.; Liang, X.; Sun, Y.; Xu, Y. Soil application of manganese sulfate effectively reduces Cd bioavailability in Cd-contaminated soil and Cd translocation and accumulation in wheat. *Science of The Total Environment* **2022**, *814*, 152765, doi:10.1016/j.scitotenv.2021.152765.
5. Sutherland, R.A.; Tack, F.M.G. Determination of Al, Cu, Fe, Mn, Pb and Zn in certified reference materials using the optimized BCR sequential extraction procedure. *Analytica Chimica Acta* **2002**, *454*, 249-257, doi:10.1016/S0003-2670(01)01553-7.
6. Lee, J.; Park, Y.-S.; Lee, H.-J.; Koo, Y.E. Microwave-assisted digestion method using diluted nitric acid and hydrogen peroxide for the determination of major and minor elements in milk samples by ICP-OES and ICP-MS. *Food Chemistry* **2022**, *373*, 131483, doi:10.1016/j.foodchem.2021.131483.
7. Wang, Z.; Tan, X.; Ma, Y.; Liu, T.; He, L.; Yang, F.; Shu, C.; Li, L.; Fu, H.; Li, B.; et al. Combining canopy spectral reflectance and RGB images to estimate leaf chlorophyll content and grain yield in rice. *Computers and Electronics in Agriculture* **2024**, *221*, 108975, doi:10.1016/j.compag.2024.108975.
8. Luo, C.; Li, T.; Huang, Y.; Liu, T.; Dong, Y. Exogenous nano-silicon enhances the ability of intercropped faba bean to alleviate cadmium toxicity and resist Fusarium wilt. *Journal of Nanobiotechnology* **2025**, *23*, 262, doi:10.1186/s12951-025-03330-0.
